# The mechanism by which miR-494-3p regulates PGC1-α-mediated inhibition of mitophagy in cardiomyocytes and alleviation of myocardial ischemia—reperfusion injury

**DOI:** 10.1186/s12872-023-03226-7

**Published:** 2023-04-21

**Authors:** Ninghui Mu, Tong Zhang, Ying Zhu, Bingtuan Lu, Qi Zheng, Jinlan Duan

**Affiliations:** 1 General Practice Department, The First People’s Hospital of Yunnan Province, Kunming, Yunnan 650032 China; 2 Geriatric Medicine Department, The First People’s Hospital of Yunnan Province, No. 157, Jinbi Road, Xishan District, Kunming, Yunnan 650032 China

**Keywords:** Myocardial ischemia–reperfusion injury, miR-494-3p, PGC1-α, Cardiomyocyte, Mitophagy

## Abstract

**Objective:**

The purpose of this study was to explore whether miR-494-3p inhibits the occurrence of mitochondrial autophagy in cardiomyocytes by inhibiting the expression of PGC1-α and to supplement the theoretical basis for the role of autophagy in cardiac injury induced by hypoxia/reperfusion (H/R).

**Methods:**

The expression of miR-494-3p was detected by RT‒qPCR, and the expression of PGC1-α, autophagy-related proteins (LC3, Beclin 1), apoptosis-related proteins (Bax and Bcl-2), PINK1/Parkin signaling pathway-related proteins (PINK1, Parkin) and mitochondrial change-related proteins (Mfn1, Mfn2, OPA1) was detected by Western blotting. The changes in mitochondrial membrane potential were detected by JC-1 staining (ΔΨm). The formation of autophagosomes was observed by transmission electron microscopy. Cell proliferation activity was detected by CCK-8, and cell apoptosis was detected by flow cytometry. A dual-luciferase gene reporter assay identified a targeted binding site between miR-494-3p and PGC1-α.

**Results:**

The results showed that miR-494-3p and PGC1-α were differentially expressed in H/R cardiomyocytes; that is, the expression of miR-494-3p was downregulated, and the expression of PGC1-α was upregulated. In addition, mitochondrial autophagy occurred in H/R cardiomyocytes. That is, LC3-II/LC3-I, Beclin 1, PINK1, and Parkin expression was upregulated, Mfn1, Mfn2, and OPA1 expression was downregulated, and the mitochondrial membrane potential was decreased. The transfection of miR-494-3p mimic can significantly improve the cell proliferation activity of cardiomyocytes and inhibit the occurrence of cardiomyocyte apoptosis and autophagy, while the transfection of miR-494-3p inhibitor has the opposite result. After transfection of the miR-494-3p mimic, treatment with autophagy inhibitors and activators changed the effects of miR-494-3p on cardiomyocyte proliferation and apoptosis. At the same time, the overexpression of PGC1-α reversed the promoting effect of miR-494-3p on cardiomyocyte proliferation and the inhibitory effect on apoptosis and autophagy.

**Conclusion:**

MiR-494-3p can target and negatively regulate the expression of PGC1-α to inhibit mitophagy in cardiomyocytes.

**Supplementary Information:**

The online version contains supplementary material available at 10.1186/s12872-023-03226-7.

## Introduction

Ischemic heart disease has become a cardiovascular disease that seriously threatens human life and health, and the number of associated deaths has increased by 22.3% in the past decade [1]. When myocardial tissue is acutely ischemic, the most effective treatment is to restore the blood supply to the dying ischemic myocardium as soon as possible. However, when blood perfusion is restored, the tissue structure and cardiac function of part of the myocardium will be affected. This can cause more serious damage, which is called myocardial ischemia reperfusion injury (MI/RI) [2]. Continuous MI/RI can cause various forms of cell death, leading to myocardial infarction, and at the same time, can cause coronary microcirculation damage to the heart [3]. In the current literature, the treatments for MI/RI, such as ischemic preconditioning, drugs that inhibit inflammation and immunity, and inhibitors of mitochondrial membrane permeability transition pore opening, cannot achieve the expected clinical results [4]. Therefore, exploring new signaling pathways and effective targets for reducing ischemia‒reperfusion injury has great significance for the prevention and treatment of MI/RI.

Mitochondrial autophagy is an intracellular self-degradation system of mitochondria that is a self-defense process that removes damaged mitochondria and maintains a healthy dynamic balance of cells under various nonphysiological stimuli [5]. Studies have shown that impaired mitochondrial autophagy and mitochondrial dysfunction are involved in the development of neurodegenerative diseases, cancer, cardiovascular diseases and other diseases [6–9]. Recently, the role of mitochondrial autophagy in MI/RI has also attracted attention. A report described rapid activation of mitochondrial autophagy in myocardial microcirculation endothelial cells induced by Parkin in a mouse model of ischemia‒reperfusion injury [10]. At the same time, studies have shown that in the process of MI/RI, the mitochondrial autophagy receptor BNIP3 can not only change the permeability of mitochondria but also induce the aggregation of autophagosomes and promote the occurrence of mitochondrial autophagy [11]. In mouse kidney and liver reperfusion injury models, inhibiting mitochondrial autophagy can protect against the injury caused by reperfusion [12, 13]. Therefore, this study explored the mechanism of mitochondrial autophagy in myocardial injury to provide an effective therapeutic target for myocardial injury.

MicroRNAs (miRNAs) are conserved single-stranded nonprotein coding RNAs with a length of approximately 21–25 nucleotides. They can inhibit the translation of messenger RNA (mRNA) to inhibit target protein-coding genes [14]. Studies have shown that miRNAs have a regulatory effect on ischemia‒reperfusion (I/R) injury [15]. For example, inhibiting miR-15 *in vivo* can protect cardiomyocytes from hypoxia-induced death [16]. Silencing miR-29 can affect the area of myocardial infarction and apoptosis by regulating the target Mcl-2 of I/R [17]. Although miRNAs are considered to be important regulators in many physiological and pathophysiological processes, their role in the regulation of mitochondrial autophagy has rarely been reported. Studies have found that miR-494-3p not only has a cardioprotective effect in MI/RI but also plays a role in mitochondrial regulation. For example, miR-494 activates the Akt pathway by simultaneously targeting proapoptotic proteins and antiapoptotic proteins, providing cardioprotection in MI/RI [18]. In addition, miR-494-3p has been shown to regulate the apoptosis of bone marrow mesenchymal stem cells in ischemic injury [19]. Moreover, miR-494-3p directly inhibits the expression of the transcriptional coactivator peroxisome proliferator activated receptor gamma coactivator 1α (PGC1-α), thereby inhibiting mitochondrial biogenesis in adipose tissue [20]. Studies have shown that PGC1-α is often induced in the process of mitochondrial biogenesis. At the same time, the expression of PGC1-α in the heart is rapidly increased under starvation or fasting conditions, and it is accompanied by the activation of mitochondrial autophagy [21]. Parkin, a key regulator of mitochondrial autophagy, can also regulate mitochondrial biogenesis by activating PGC1-α transcription [22]. PGC1-α plays an important role in the occurrence of mitochondrial autophagy, and the regulation of PGC1-α by miR-494-3p is also a potential cause affecting the occurrence of mitochondrial autophagy. The role of this regulatory relationship in MI/RI remains unknown. Therefore, this study investigated whether miR-494-3p alleviates myocardial ischemia‒reperfusion injury by regulating PGC1-α to inhibit mitochondrial autophagy.

## Materials and methods

### Establishment of the cell H/R cell model [23]

The rat myocardial cell line (H9C2, GNR5) was purchased from Cell Center, Chinese Academy of Sciences, Shanghai. When the confluence rate of H9C2 cardiomyocytes in Dulbecco’s Modified Eagle Medium (DMEM, MA0212, meilunbio, Dalian, China) containing 15% fetal bovine serum (10,099,141 C, Gibco, Australian) reached 80–90%, the H9C2 cardiomyocyte medium was replaced with Earle’s Balanced Salt Solution (EBSS, PB180337, Pricella, Wuhan, China) medium, placed in a humid hypoxia chamber under the control of 95% N_2_ + 5% CO_2_, and incubated for 6 h. Then, the H9C2 cardiomyocyte medium was replaced with high glucose DMEM containing 15% fetal bovine serum, and reoxygenated culture conditions (95% Air + 5% CO_2_) were controlled for 3 h.

### Cell transfection

miR-494-3p inhibitors (5’-AGAGGUUUCCCGUGUAUGUUUCA-3’) and miR-494-3p mimics (Forward : 5’-UGAAACAUACACGGGAAACCUC-3’; Reverse: 5’-GGUUUCCCGUGUAUGUUUCAAU-3’) and their relative controls (NC inhibitors and NC mimics) were obtained from Thermo Fisher Scientific (Waltham, MA, USA). The PGC1-α overexpression plasmid (OE-PGC1-α) was constructed using pcDNA3.1 vector (V790-20, Addgene, USA). An empty plasmid cloning pcDNA3.1 vector used as a control (NC-OE). Cell transfection was performed according to Lipofectamine 2000 (11,668,019, Thermo Fisher Scientific, USA) instructions. In simple terms, cardiomyocytes (1 × 10^5^) are spread into a 6-well plate. Diluted DNA was added with diluted Lipofectamine®2000 reagent (1:1 ratio) and incubated at room temperature for 5 min. Then add the DNA-lipid complex to the cells. The transfected cells were analyzed after incubation at 37 ℃ for 48 h.

### Real-time quantitative polymerase chain reaction (RT‒qPCR)

After cell collection, total RNA was extracted by TRIzol Reagent (R0016, Beyotime, Shanghai, China). RNA samples were reversed transcription using the TaqMan® MicroRNA reverse Transcription kit (4,366,596, Applied Biosystems, Foster City, USA). qPCR was performed using SYBR qPCR Master Mix (Q311-02, Vazyme, Nanjing, China). The primers used in this study were as follows: miR-494-3p Forward: 5’-GCGTGAAACATACACGGGAA-3’; Reverse: 5’-AGTGCAGGGTCCGAGGTATT-3’; U6 Forward: 5’- GCTTCGGCAGCACATATACTAAAAT − 3’; Reverse: 5’- CGCTTCACGAATTTGCGTGTCAT − 3’. U6 was the load control group of miR-494-3p, and the data were analyzed by the 2^−ΔΔCt^ method.

### Western blot analysis

Total protein was extracted from cells with RIPA lysis buffer (R0010, Solarbio, Beijing, China). A bicinchoninic acid (BCA) protein assay kit (P0012, Beyotime, Shanghai, China) was used to determine the protein concentration. The protein was detected by sodium dodecyl sulfat-polyacrylamide gel electrophoresis and electrically imprinted on the membrane. Membranes were incubated with primary antibodies PGC1-α (1:1000, ab106814, Abcam, UK), LC3 (1:1000, ab63817, Abcam, UK), Beclin 1 (1:2000, ab207612, Abcam, UK), PINK1 (1:1000, ab300623, Abcam, UK), Parkin (1:2000, ab77924, Abcam, UK), Mfn1 (1:1000, ab221661, Abcam, UK), Mfn2 (1:2000, ab205236, Abcam, UK), OPA1 (1:2000, ab42364, Abcam, UK), GAPDH (1:2000, ab8245, Abcam, UK) at 4℃ overnight, then incubated with horseradish peroxidase (HRP) labeled secondary antibody (1:2000, ab97051, Abcam, UK). Enhanced chemiluminescence (ECL) kit (WP20005, Thermo Fisher Scientific, Waltham, USA) was used for color development. Finally, the gray values of protein bands were analyzed by ImageJ software.

### Dual luciferase reporter gene

The biological information database TargetScan was used to predict the targeting binding sites of miR-494-3p and PGC1-α. The binding site sequence of miR-494-3p and PGC1-α 3’-UTR and its mutant sequence were inserted downstream of the firefly luciferase gene to construct the expression vector. HEK 293T cells were cotransfected with miR-494-3p mimic/NC-mimic and pmirGLO-PGC1-α 1 wild-type (WT)/mutant (MUT) recombinant plasmids, and cells were collected 48 h after transfection. Luciferase activity was detected using a Dual Luciferase Reporter Gene Assay Kit (RG027, Beyotime, Shanghai, China).

### Cell proliferation activity detected by CCK-8

The H9C2 cell concentration was adjusted to 1 × 10^5^/mL, and the cell suspension was inoculated in a 96-well plate at a volume of 150 µL/well and incubated at 37 ℃ and 5% CO_2_ for 24 h. The cells were treated with drugs or transfected according to different groups, then CCK-8 complete medium solution (C0037, Beyotime, Shanghai, China) was added and incubated for 90 min in the same temperature box. The optical density at λ = 450 nm was measured with a microplate reader.

### Apoptosis detected by flow cytometry

H9C2 cardiomyocytes were rinsed twice with sterile Phosphate-Buffered Saline (PBS) solution precooled at 4 °C. Then, 500 µL binding buffer was added to each well, and 10 µL Annexin V-FITC was added and mixed gently for 3 min. Next, 10 µL 20 µg/mL PI solution (40302ES20, Yeasen Biotechnology, Shanghai,China) was mixed and incubated at room temperature, protected from light, for 15 min. Apoptotic cells were detected and analyzed by flow cytometry.

### Transmission electron microscopy observation of autophagosomes

H9C2 cells were removed after reoxygenation (3 h), the supernatant was discarded with a pipette, and the cells were washed twice with PBS. The cells were transferred to a 15 mL centrifuge tube at a speed of 1000 rpm for 5 min. The supernatant was discarded and the cells were suspended with 1 mL PBS, transferred to a 1.5 mL eppendorf (EP) tube, and centrifuged at 1000 rpm for 5 min. After the supernatant was removed, 1 mL 2.5% glutaraldehyde was slowly added and fixed at 4℃ for 2 h. The precooled PBS was rinsed 3 times, and the acetone was dehydrated. At room temperature, it was embedded with 1% tomahawk acid and epoxy resin for 2 h. After drying, the blocks were repaired and sliced into ultrathin sections. Uranium dioxy acetate and lead cerate were stained for 15 min and 5 min, respectively. Ultrastructural changes and autophagosomes of H9C2-treated cells were observed under transmission electron microscopy.

### JC-1 staining

The JC-1 Mitochondrial Membrane Potential Assay Kit (C2006, Beyotime, Shanghai, China) was used to detect changes in mitochondrial membrane potential in cardiomyocytes. In simple terms, cardiomyocytes were seeded in 6-well plates, and after different treatments, the cell culture medium was removed and the cells were washed once with PBS. Then, 1 ml of cell culture medium and 1 ml of JC-1 staining working solution was added and mixed well, and the cells were incubated in a 37 °C cell culture incubator for 20 min. After incubation, the cells were washed twice with JC-1 staining buffer (1X), 2 ml of cell culture medium was added, and the cells were observed under a fluorescence microscope.

**Fig. 1:**
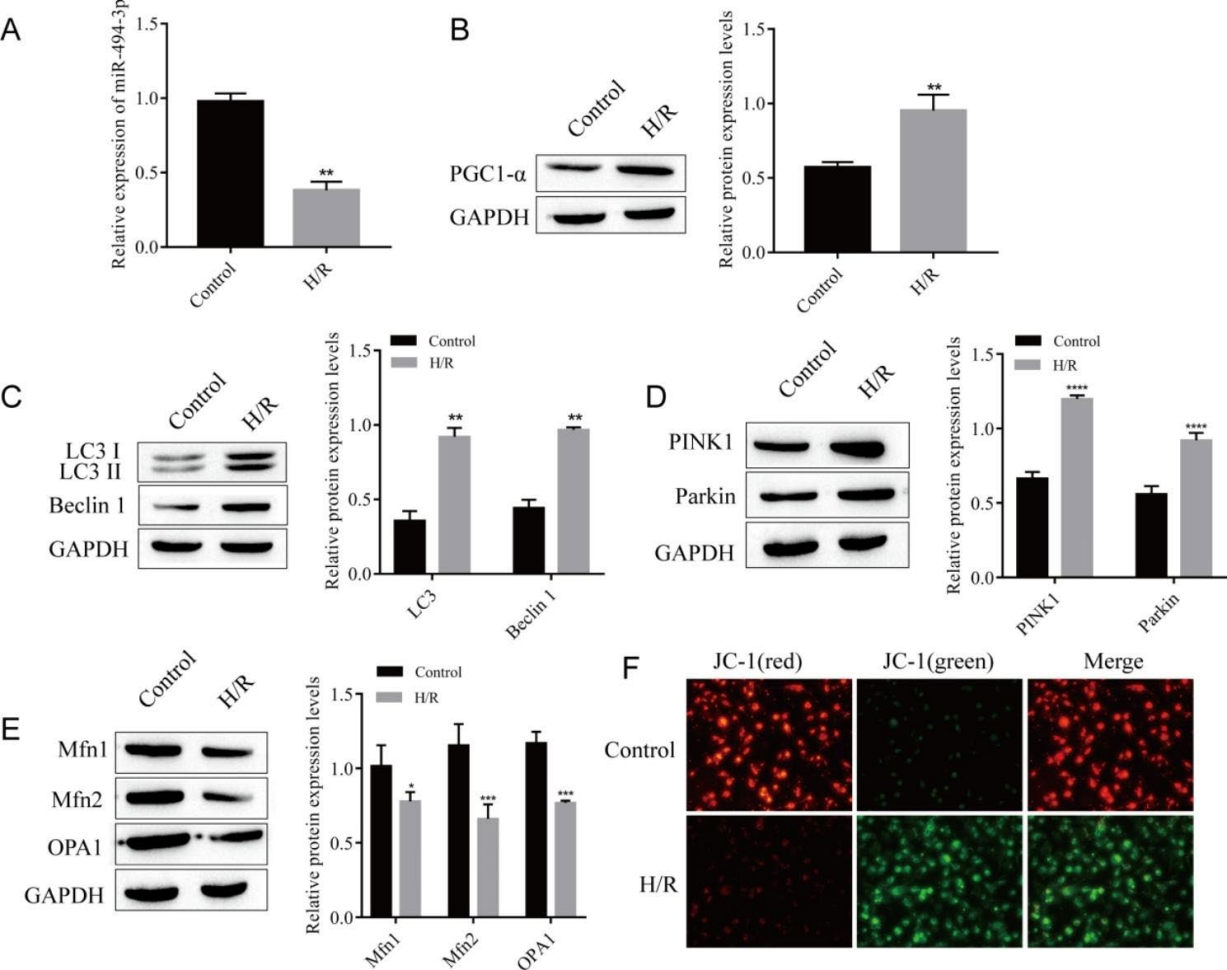
**Mitochondrial autophagy and differential expression of miR-494-3p and PGC1-α were observed in H/R cardiomyocytes** (A) The expression of miR-494-3p was detected by RT‒qPCR. (B) The expression of PGC1-α was detected by Western blotting. (C) The expression of the autophagy-related proteins LC3 and Beclin 1 was detected by Western blotting. (D) The expression of PINK1 and Parkin was detected by Western blotting. (E) The expression of Mfn1, Mfn2 and OPA1 was detected by Western blotting. (F) Changes in ΔΨm were detected by JC-1 staining. ^*^P < 0.05, ^**^P < 0.01, ^*^P < 0.001, ^****^P < 0.0001 vs. control. n = 3, all experiments were repeated three times.

### Statistical analysis

The SPSS 17.0 software package was used for statistical analysis, and all data are expressed as the mean ± standard deviation (mean ± SD). Data between two groups were compared using Student’s *t* test, and one-way analysis of variance (ANOVA) was used to compare data between multiple groups.

**Fig. 2:**
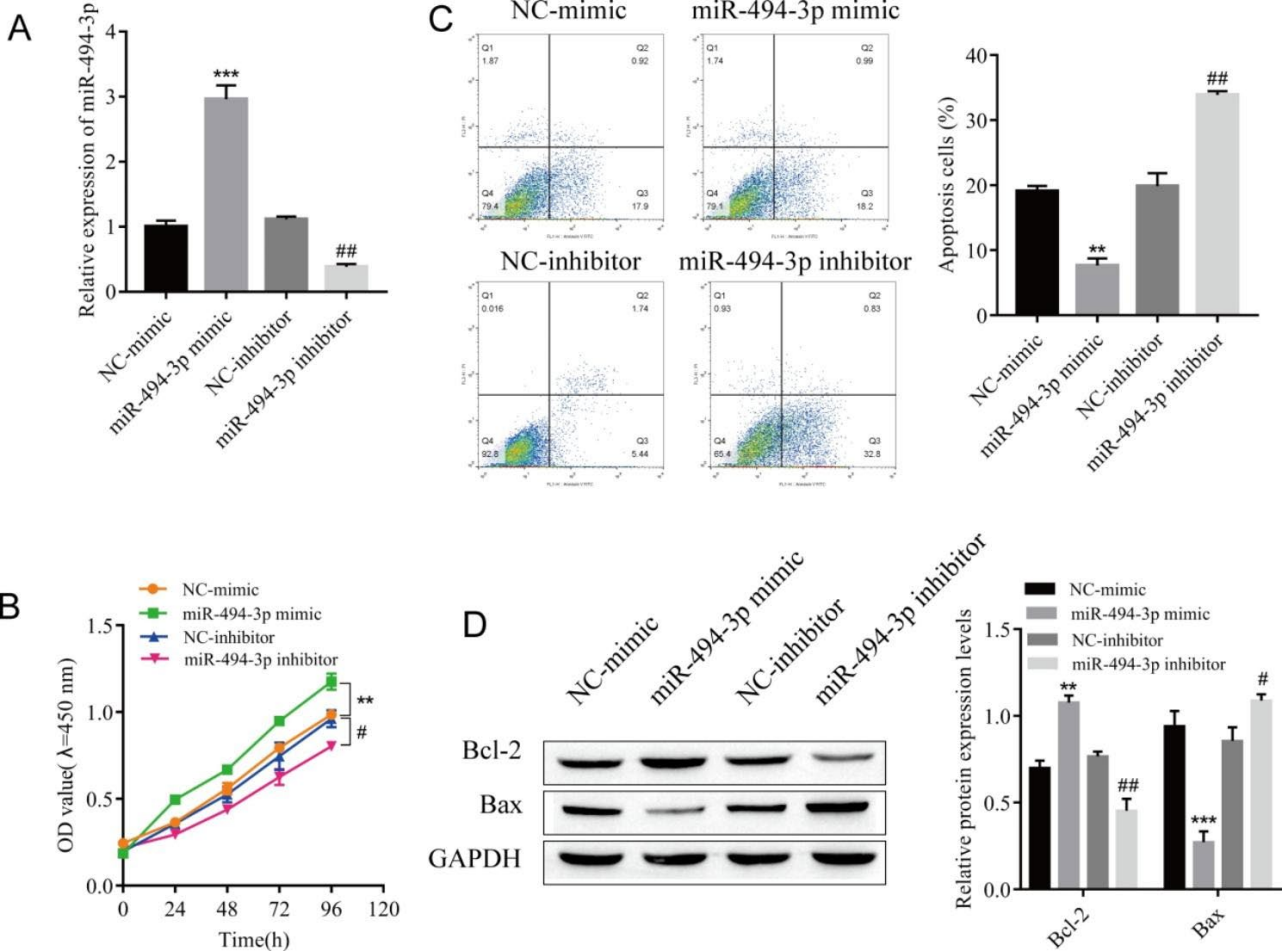
**Effects of miR-494-3p on the proliferation and apoptosis of cardiomyocytes** (A) The expression of miR-494-3p was detected by RT‒qPCR. (B) CCK-8 assay of the proliferative activity of cardiomyocytes. (C) Apoptosis of cardiomyocytes was detected by flow cytometry. (D) The expression of the apoptosis-related proteins Bax and Bcl-2 was detected by Western blotting. ^**^P < 0.01, ^***^P < 0.001 vs. NC-mimic; ^#^*P* < 0.05, ^##^*P* < 0.01 vs. NC-inhibitor. n = 3, all experiments were repeated three times.

## Results

### Mitochondrial autophagy and differential expression of mir-494-3p and PGC1-α were observed in H/R cardiomyocytes

H9C2 cells cultured in normal oxygen were taken as the control group (control), and the hypoxia and reoxygenation cells were taken as the experimental group (H/R). RT‒qPCR detection showed that miR-494-3p expression was downregulated in the H/R group compared with the control group (Fig. 1A). Meanwhile, Western blot analysis showed that compared with the control group, the expression of PGC1-α was significantly upregulated in the H/R group (Fig. 1B). In addition, we detected autophagy-associated proteins, and the expression of LC3 and Beclin 1 was upregulated in the H/R group compared with the control group (Fig. 1C). We further determined that mitochondrial autophagy occurred in H/R cells. We found that the expression of PINK1 and Parkin proteins in the PINK1/Parkin signaling pathway, which is closely related to mitochondrial autophagy, was significantly upregulated in the H/R group (Fig. 1D), while the expression of Mfn1, Mfn2 and OPA1 proteins, which regulate mitochondrial fusion, division and autophagy, was downregulated (Fig. 1E). Finally, the mitochondrial membrane potential (ΔΨm) was observed by JC-1 staining. The results showed that the red fluorescence was stronger and the green fluorescence was weaker in the control group, and the ΔΨm was higher. After H/R treatment, the green fluorescence was enhanced, the red fluorescence was weakened, and the ΔΨm was decreased (Fig. 1F).

**Fig. 3:**
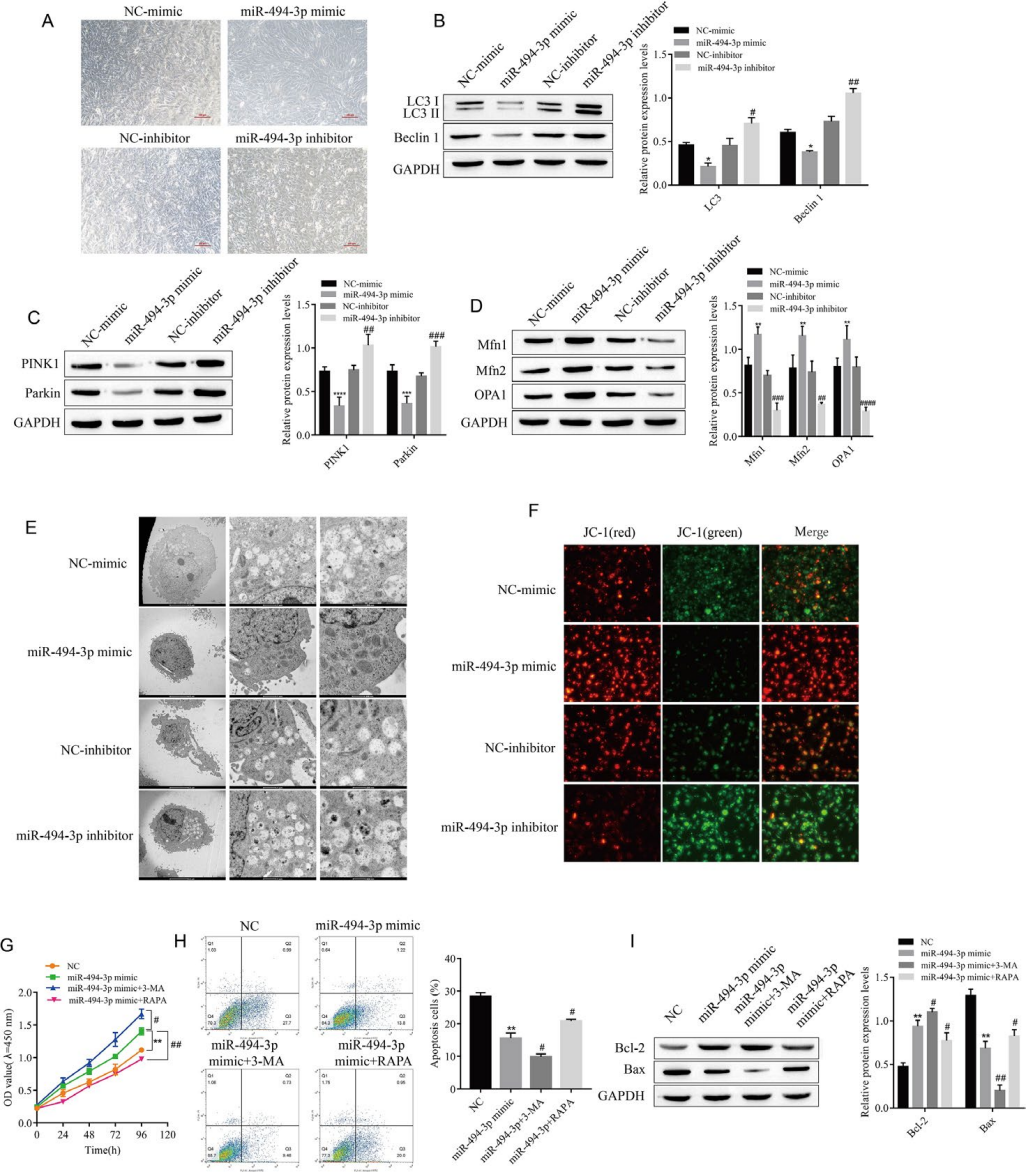
**miR-494-3p affects the proliferation and apoptosis of cardiomyocytes by affecting mitochondrial autophagy** (A) Morphological observation of cardiomyocytes, scale bar = 100 μm. (B) The expression of the autophagy-related proteins LC3 and Beclin 1 was detected by Western blotting. (C) The expression of PINK1 and Parkin was detected by Western blotting. (D) The expression of Mfn1, Mfn2 and OPA1 was detected by Western blotting. (E) The formation of autophagosomes was observed by transmission electron microscopy, scale bar = 5 μm/1 µm/500 nm. (F) Changes in ΔΨm were detected by JC-1 staining. (G) CCK-8 assay of the proliferative activity of cardiomyocytes. (H) Apoptosis of cardiomyocytes was detected by flow cytometry. (I) The expression of the apoptosis-related proteins Bax and Bcl-2 was detected by Western blotting. ^*^P < 0.05, ^**^P < 0.01, ^***^P < 0.001, ^****^P < 0.0001 vs. NC-mimic or NC; ^#^*P* < 0.05, ^##^*P* < 0.01, ^###^*P* < 0.001, ^####^*P* < 0.0001 vs. NC-inhibitor or miR-494-3p mimic. n = 3, all experiments were repeated three times.

**Fig. 4:**
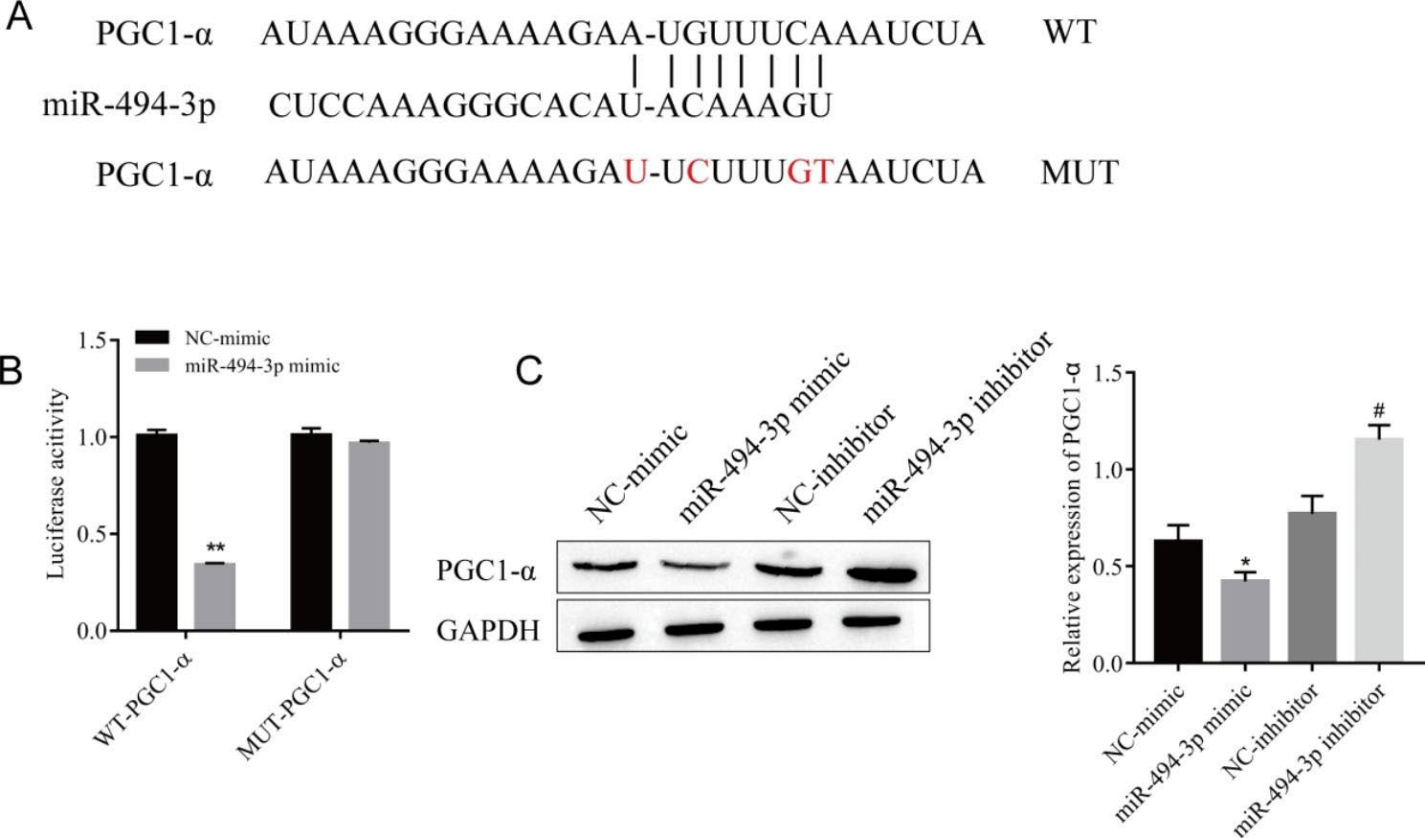
**miR-494-3p targets and regulates the expression of PGC1-α** (A) Prediction of targeted binding sites. (B) Double luciferase gene reporter assay. (C) Western blotting was used to detect the expression of PGC1-α. ^*^P < 0.05, ^**^P < 0.01 vs. NC-mimic; ^#^*P* < 0.05 vs. NC-inhibitor. n = 3, all experiments were repeated three times.

**Fig. 5:**
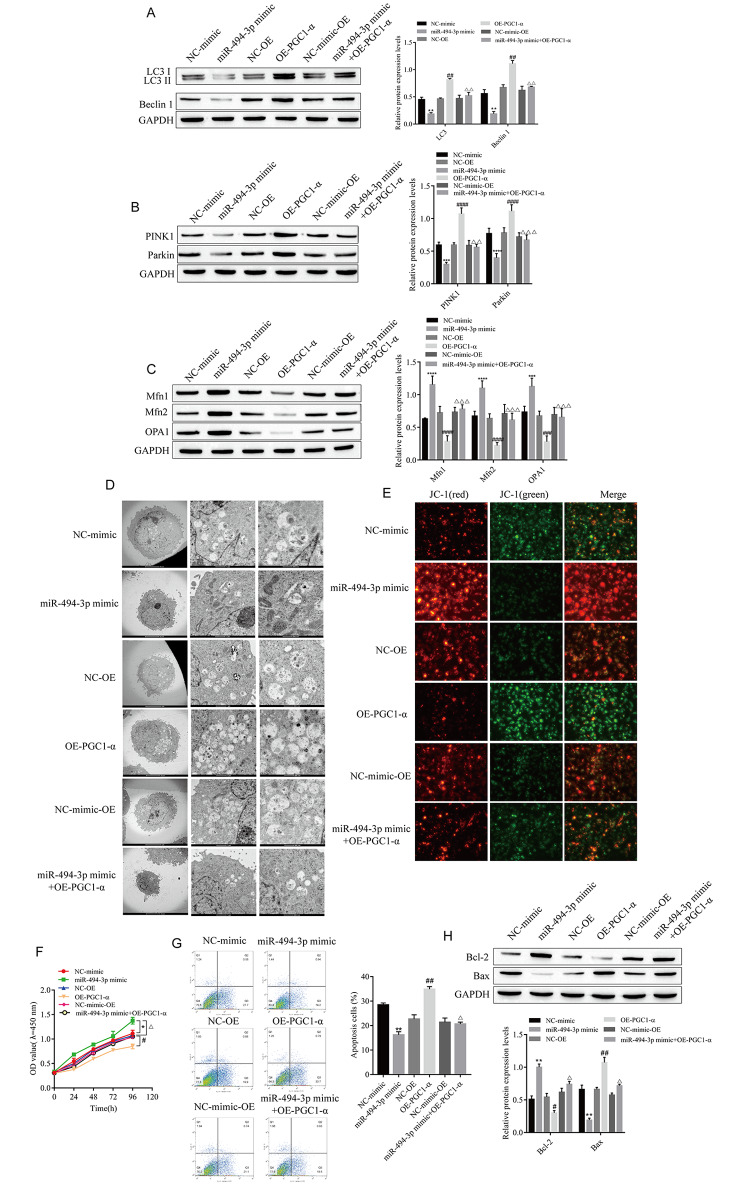
**miR-494-3p affects mitochondrial autophagy by regulating PGC1-α** (A) The expression of the autophagy-related proteins LC3 and Beclin 1 was detected by Western blotting. (B) The expression of PINK1 and Parkin was detected by Western blotting. (C) The expression of Mfn1, Mfn2 and OPA1 was detected by Western blotting. (D) The formation of autophagosomes was observed by transmission electron microscopy, scale bar = 5 μm/1 µm/500 nm. (E) Changes in ΔΨm were detected by JC-1 staining. (F) CCK-8 assay of the proliferative activity of cardiomyocytes. (G) Apoptosis of cardiomyocytes was detected by flow cytometry. (H) The expression of the apoptosis-related proteins Bax and Bcl-2 was detected by Western blotting. ^*^P < 0.05, ^**^P < 0.01, ^***^P < 0.001, ^****^P < 0.0001 vs. NC-mimic; ^#^*P* < 0.05, ^##^*P* < 0.01, ^###^*P* < 0.001, ^####^*P* < 0.0001 vs. NC-OE; ^Δ^*P* < 0.05, ^ΔΔ^*P* < 0.01, ^ΔΔΔ^*P* < 0.001 vs. miR-494-3p mimic. n = 3, all experiments were repeated three times.

### Effects of miR-494-3p on the proliferation and apoptosis of cardiomyocytes

After the differential expression of miR-494-3p in cardiomyocytes was observed, we further confirmed the effect of miR-494-3p on the proliferation and apoptosis of cardiomyocytes. The proliferation and apoptosis of myocardial cells were observed after transfection with miR-494-3p mimic or miR-494-3p inhibitor. First, the expression of miR-494-3p was detected by RT‒qPCR to confirm the transfection efficiency. The expression of miR-494-3p in the miR-494-3p mimic group was significantly increased compared with that in the NC-mimic group, and compared with that in the NC-inhibitor group, miR-494-3p expression in the miR-494-3p inhibitor group was significantly downregulated (Fig. 2A), indicating successful transfection. Meanwhile, the CCK-8 results showed that the proliferation activity of myocardial cells was enhanced after transfection with the miR-494-3p mimic, while this activity was significantly inhibited after transfection with the miR-494-3p inhibitor (Fig. 2B). Similarly, compared with the NC-mimic group, the apoptosis of myocardial cells in the miR-494-3p mimic group was weakened, the expression of apoptotic protein Bax was decreased, and the expression of anti-apoptotic protein Bcl-2 was increased (Fig. 2C-D). Compared with the NC-inhibitor group, the apoptosis of myocardial cells in the miR-494-3p inhibitor group was increased, the expression of the apoptotic protein Bax was increased, and the expression of the anti-apoptotic protein Bcl-2 was decreased (Fig. 2C-D).

### Mir-494-3p affects the proliferation and apoptosis of cardiomyocytes by affecting mitochondrial autophagy

Mitochondrial autophagy was observed in H/R cardiomyocytes, so we sought to determine whether the effect of miR-494-3p on the proliferation and apoptosis of cardiomyocytes was also related to mitochondrial autophagy. First, the effects of miR-494-3p mimic and miR-494-3p inhibitor on the morphological changes of H/R cardiomyocytes after transfection were observed. The results showed that the growth of cardiomyocytes in the miR-494-3p mimic group was the best, and the cells were spindled and neatly arranged. However, the miR-494-3p inhibitor group showed the worst myocardial cell differentiation and polygonal cell clusters (Fig. 3A). Then, the effect of miR-494-3p on mitochondrial autophagy in cardiomyocytes was observed. The results showed that the expression levels of LC3, Beclin 1, Pink1, and Parkin were downregulated and the expression levels of Mfn1, Mfn2, and OPA1 were upregulated in the miR-494-3p mimic group compared with the NC-mimic group. However, the transfection of miR-494-3p inhibitor had the opposite effect on the expression of the above proteins (Fig. 3B-D). In addition, autophagosome formation was observed by transmission electron microscopy and mitochondrial membrane potential was observed by JC-1 staining. We found that autophagosomes decreased and the ΔΨm increased in the miR-494-3p mimic group compared with the NC-mimic group. At the same time, compared with the NC-inhibitor group, autophagosomes increased and the ΔΨm decreased in the miR-494-3p inhibitor group (Fig. 3E-F). We further confirmed that autophagy is an important factor in the effect of miR-494-3p on the proliferation and apoptosis of cardiomyocytes. After transfecting the miR-494-3p mimic into H/R cardiomyocytes, the cells were treated with an autophagy inhibitor (3-MA) or autophagy activator (RARP). The CCK-8 test results showed that the autophagy inhibitor 3-MA enhanced the promoting effect of miR-494-3p on the proliferation of cardiomyocytes, while the autophagy activator RARP weakened the promoting effect of miR-494-3p on the proliferation of cardiomyocytes (Fig. 3G). Similarly, 3-MA enhanced the inhibitory effect of miR-494-3p on myocardial cell apoptosis, while PARP weakened the inhibitory effect of miR-494-3p on myocardial cell apoptosis. That is, compared with the miR-494-3p mimic group, cell apoptosis was decreased, the expression of the apoptotic protein Bax was downregulated, and the expression of the antiapoptotic protein Bcl-2 was upregulated in the miR-494-3p mimic+ 3-MA group. Meanwhile, apoptosis was increased, the expression of the apoptotic protein Bax was upregulated, and the expression of the anti-apoptotic protein Bcl-2 was downregulated in the miR-494-3p mimic+ RARP group (Fig. 3H-I).

### Mir-494-3p targets to regulate the expression of PGC1-α

Through bioinformatics software, we predicted that there was a targeted binding site between miR-494-3p and PGC1-α (Fig. 4A), and this relationship was further confirmed by a double luciferase gene reporter assay. The relative fluorescence intensity of PGC1-α in the miR-494-3p mimic + PGC1-α-3’UTR WT group was significantly lower than that in the NC-mimic + PGC1-α-3’UTR WT group (Fig. 4B). After mutation of miR-494-3p binding sites in the PGC1-α 3’UTR, the inhibitory effect of the miR-494-3p mimic on reporter genes disappeared. That is, the relative fluorescence intensity of PGC1-α in the miR-494-3p mimic + PGC1-α-3’UTR MUT group was similar to that in the NC-mimic +  PGC1-α-3’UTR MUT group (Fig. 4B). Western blot analysis confirmed that PGC1-α protein expression was downregulated in the miR-494-3p mimic group compared with the NC-mimic group. At the same time, the expression level of PGC1-α protein in the miR-494-3p inhibitor group was significantly upregulated compared with that in the NC-inhibitor group (Fig. 4C). This suggests that miR-494-3p negatively regulates the expression of PGC1-α.

### MiR-494-3p affects mitochondrial autophagy by regulating PGC1-α

To confirm that miR-494-3p affects mitochondrial autophagy by regulating PGC1-α and the proliferation and apoptosis of cardiomyocytes, PGC1-α was overexpressed in cardiomyocytes and cotransfected with miR-494-3p mimic and PGC1-α overexpression plasmid. Autophagy formation, cell proliferation and apoptosis were observed. The results showed that the expression levels of LC3, Beclin 1, PINK1 and Parkin were upregulated and the expression levels of Mfn1, Mfn2 and OPA1 were downregulated after the overexpression of PGC1-α. Moreover, overexpression of PGC1-α reversed the inhibitory effects of the miR-494-3p mimic on LC3, Beclin 1, PINK1, and Parkin and promoted the expression of Mfn1, Mfn2, and OPA1 (Fig. 5A-C). We also found that the overexpression of PGC1-α increased autophagosome generation and decreased the ΔΨm, which was contrary to the effect of transfection of the miR-494-3p mimic alone. At the same time, we overexpressed PGC1-α while transfecting the miR-494-3p mimic and found that the overexpression of PGC1-α reversed the inhibitory effect of the miR-494-3p mimic on autophagosome formation and the promoting effect of the miR-494-3p mimic on the increase in the ΔΨm (Fig. 5D-E). The CCK-8 results showed that the proliferation activity of cardiomyocytes decreased after the overexpression of PGC1-α, and the promoting effect of miR-494-3p on cell proliferation activity was reversed after the overexpression of PGC1-α (Fig. 5F). Flow cytometry showed that the overexpression of PGC1-α increased myocardial apoptosis, upregulated the expression of the apoptotic protein Bax, downregulated the expression of the antiapoptotic protein Bcl-2, and reversed the inhibitory effect of miR-494-3p on apoptosis (Fig. 5G-H).

## Discussion

Reperfusion during acute myocardial ischemia is an effective method to save the heart, and MI/RI is caused when the ischemic myocardial tissue resumes blood perfusion [24]. This study identified a new molecular regulatory mechanism of MI/RI, namely, miR-494-3p can inhibit the mitochondrial autophagy transition of cardiomyocytes by regulating PGC1-α, thus alleviating the occurrence of myocardial ischemia‒reperfusion injury and providing a new perspective for the prevention and treatment of MI/RI.

Mitochondrial autophagy, a subtype of autophagy, is a mechanism leading to lysosomal degradation of cellular components [25, 26]. Studies have shown that autophagy of cardiomyocytes is crucial for cardiomyocytes to maintain their normal state and function, and autophagy plays a “double-edged sword” role in myocardial ischemia and reperfusion. Sybers et al. [27] were the first scholars to discover the existence of autophagy during I/R injury, which has been confirmed by subsequent studies. Decker et al. [28] established the Langendorff perfusion rabbit model and found that autophagy significantly increased during reperfusion after 40 min of myocardial ischemia. Matsui et al. [29] also found that in the area of myocardial ischemia, autophagy increased significantly. In this study, by constructing an H/R cell model, it was found that autophagy also occurred in H/R cardiomyocytes compared with normal cardiomyocytes, and it was determined that autophagy in myocardial I/R damages cardiomyocytes. LC3 protein plays a key role in the formation of autophagosomes. LC3 contains type I and type II, and the content of LC3-II is closely related to autophagy activity [30, 31]. In this study, the expression levels of Beclin 1 and LC3-II/LC3-I were detected in H9C2 cardiomyocytes of the control group and H/R group. The final test results showed that compared with the control group, the hypoxia/reoxygenation group had significantly increased protein expression levels of Beclin 1 and LC3-II/LC3-I. The PINK1/Parkin pathway is the clearest pathway for mitochondrial autophagy [32]. When the mitochondrial membrane potential is depolarized, PINK1 attached to the mitochondrial outer membrane will recruit Parkin protein in the cytoplasm and migrate to the mitochondrial outer membrane, and Parkin can recruit autophagy receptor p62 to promote the formation of autophagy lysosomes [33, 34]. We found that the expression of PINK1 and Parkin was upregulated, the mitochondrial membrane potential was decreased, and the expression of Mfn1, Mfn2, and OPA1 proteins associated with mitochondrial fusion, division, and autophagy was downregulated in H/R cardiomyocytes. This indicates that H/R is accompanied by autophagy of myocardial cell mitochondria.

As an important regulatory factor, miRNA has also been reported to play a role in the regulation of mitochondrial autophagy, and it is related to the occurrence of I/R injury. For example, miRNA-410 is significantly upregulated in the mouse I/R model, and its interaction with HMGB1 leads to deterioration of mitochondrial function and mitochondrial autophagy [35]. MiRNA-330-3p inhibits mitochondrial autophagy induced by phosphoglycerate mutant enzyme family member 5 and reduces liver ischemia‒reperfusion injury [36]. At the same time, studies have shown that miRNA-494-3p plays a protective role in myocardial ischemia‒reperfusion injury by inhibiting BRD4 [37]. MiR-494-3p mediates the PGC1-α-TFAM signaling pathway by alleviating mitochondrial dysfunction and the resulting hepatocyte apoptosis [38]. In this study, miR-494-3p was also found to play a protective role in myocardial ischemia‒reperfusion injury. MiR-494-3p can promote the proliferation and inhibit the apoptosis of cardiomyocytes, as well as inhibit excessive autophagy of cardiomyocytes, thus negatively targeting PGC1-α.

## Conclusion

In this study, we found downregulated expression of miR-494-3p, upregulated expression of PGC1-α, and excessive autophagy in H/R cardiomyocytes. We demonstrated that miR-494-3p plays a protective role in myocardial ischemia reperfusion injury by inhibiting mitochondrial autophagy in cardiomyocytes by regulating PGC1-α. This study provides a new interpretation of the correlation between mitochondrial autophagy and MI/RI and provides ideas for the selection of new therapeutic targets for myocardial ischemia‒reperfusion injury.

## Electronic supplementary material

Below is the link to the electronic supplementary material.

Additional File:

## Data Availability

The datasets used and/or analyzed during the current study are available from the corresponding author upon reasonable request.
