# Supplementary material for: The mechanism by which miR-494-3p regulates PGC1-α-mediated inhibition of mitophagy in cardiomyocytes and alleviation of myocardial ischemia—reperfusion injury
Source: BMC Cardiovasc Disord. 2023 Apr 21;23:204. doi: 10.1186/s12872-023-03226-7 (PMC10122381; doi:10.1186/s12872-023-03226-7)
Supplement: Supplementary file 1 — Additional File: [file 12872_2023_3226_MOESM1_ESM.pdf]

Figure 1B

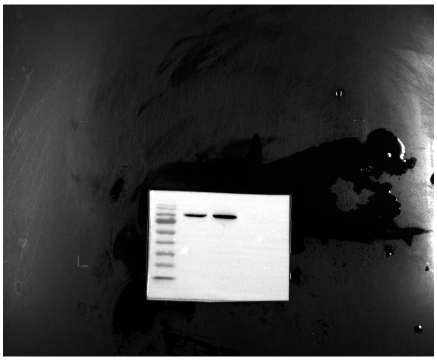

PGC1-α

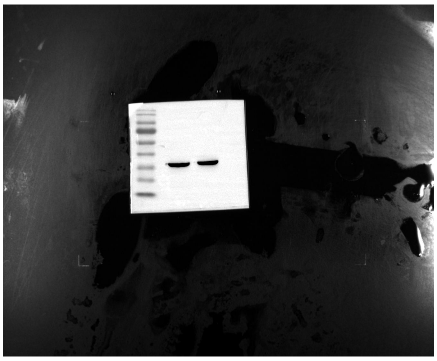

GAPDH

Figure 1C

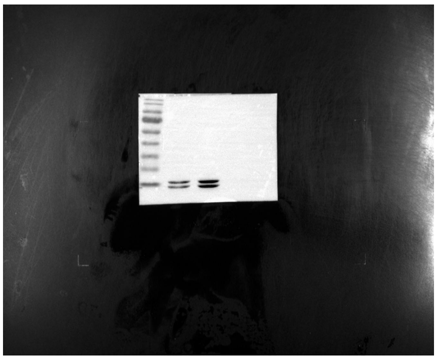

LC3

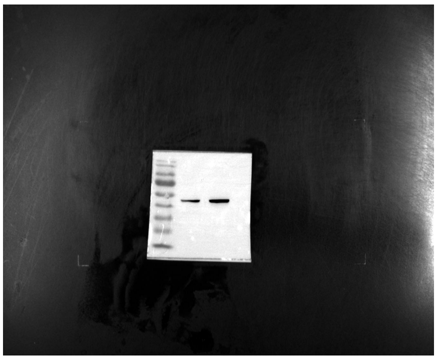

Beclin1

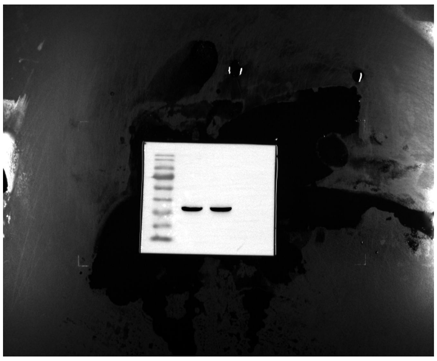

GAPDH

Figure 1D

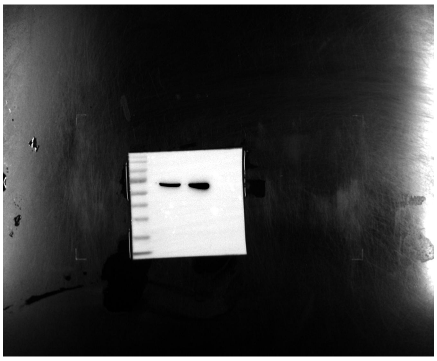

PINK1

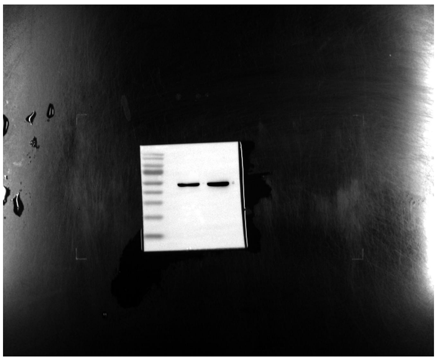

Parkin

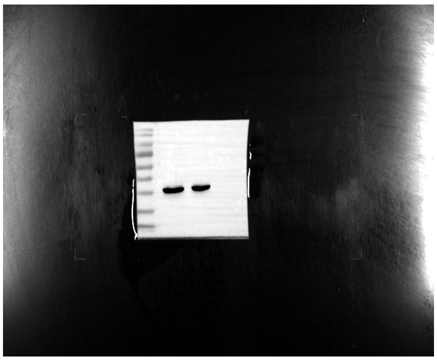

GAPDH

Figure 1E

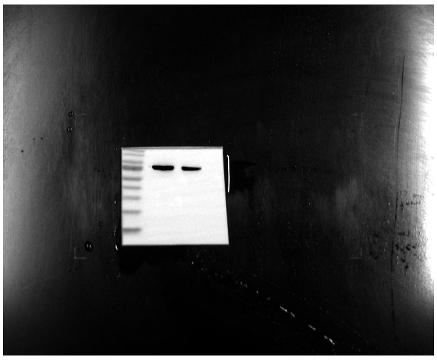

Mfn1

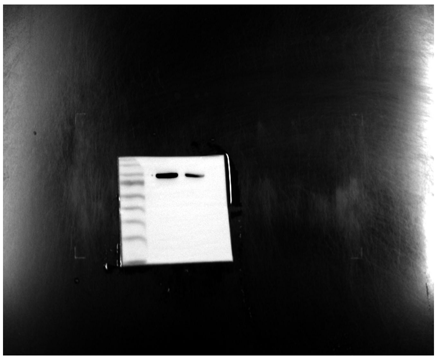

Mfn2

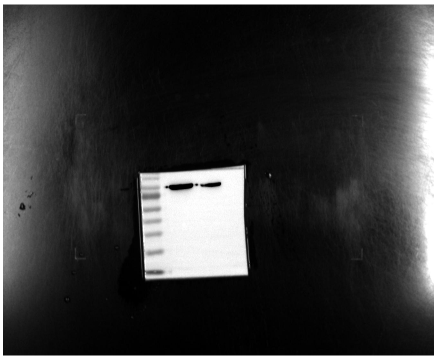

OPA1

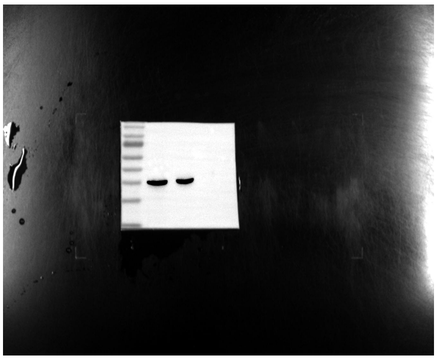

GAPDH

Figure 2D

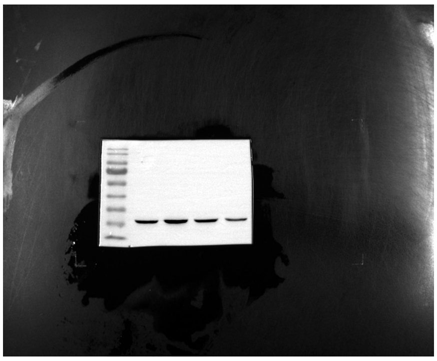

Bcl-2

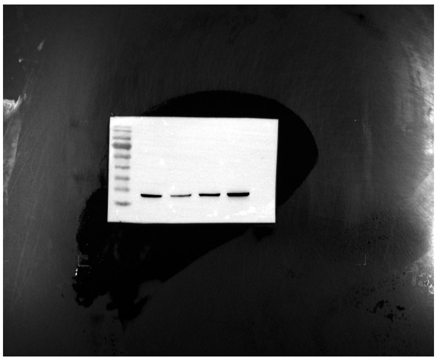

Bax

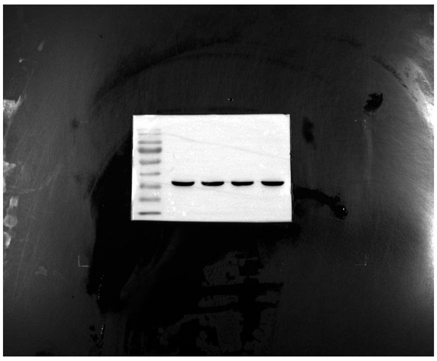

GAPDH

Figure 3B

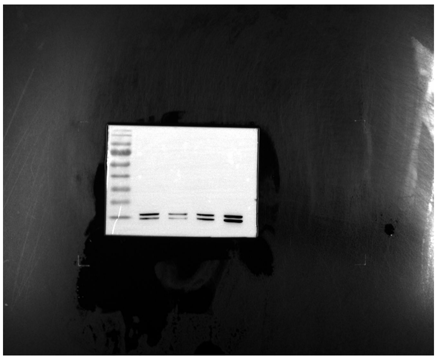

LC3

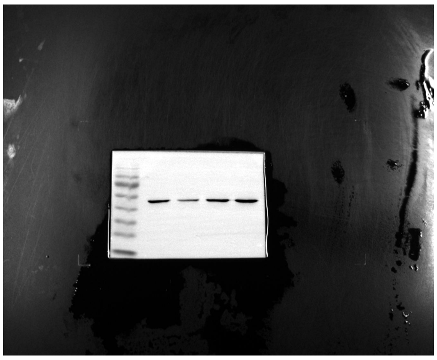

Beclin 1

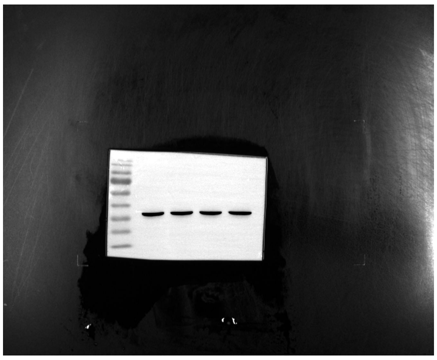

GAPDH

Figure 3C

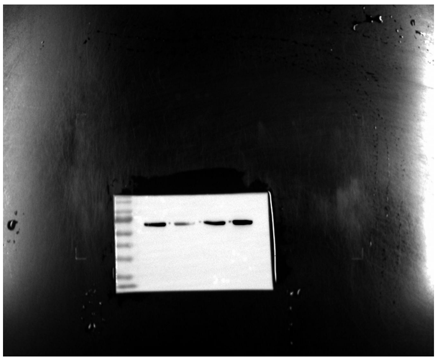

PINK1

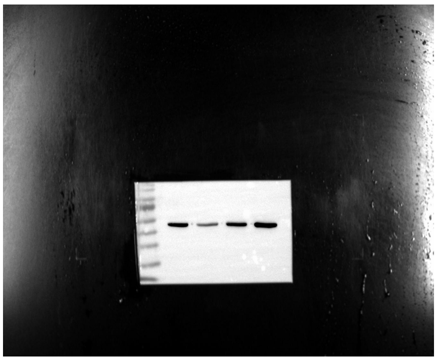

Parkin

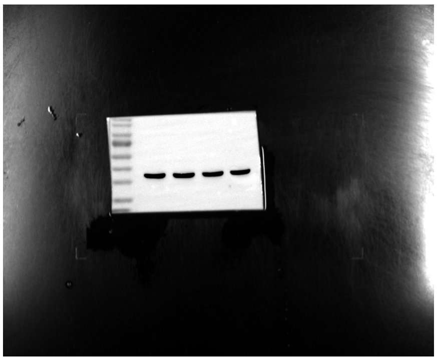

GAPDH

Figure 3D

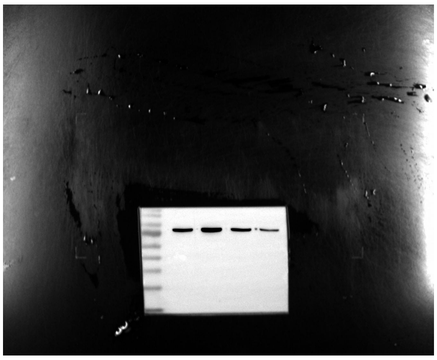

Mfn1

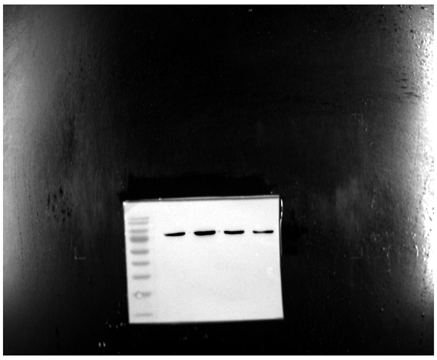

Mfn2

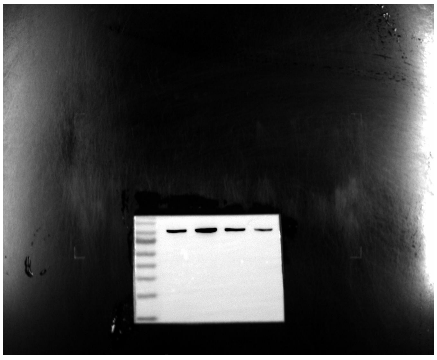

OPA1

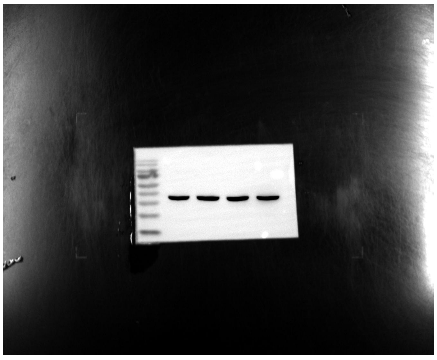

GAPDH

Figure 3I

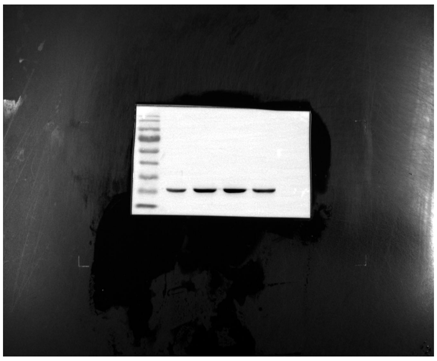

Bcl-2

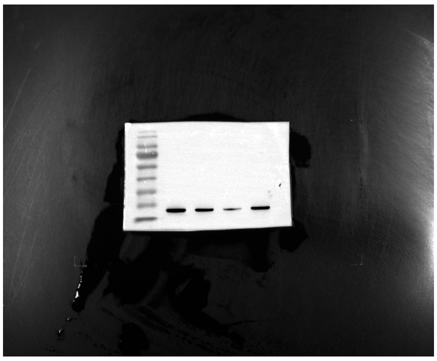

Bax

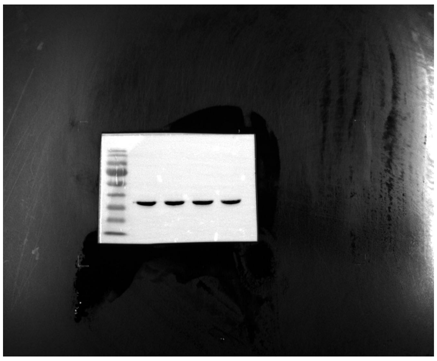

GAPDH

Figure 4C

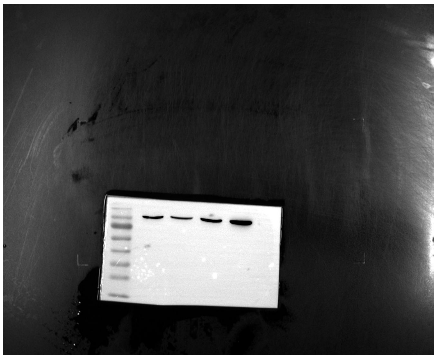

PGC1-α

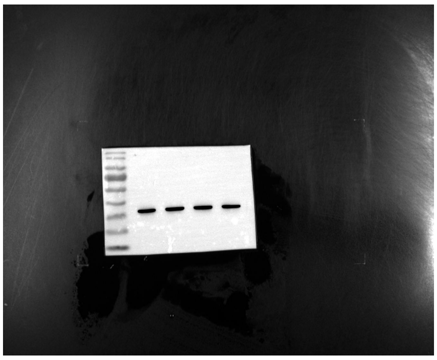

GAPDH

Figure 5A

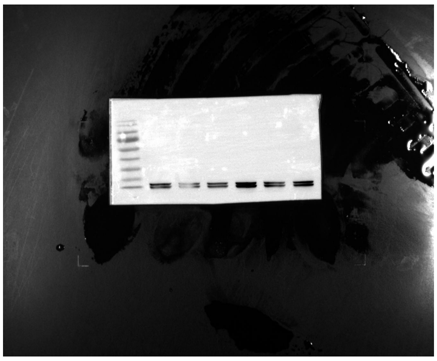

LC3

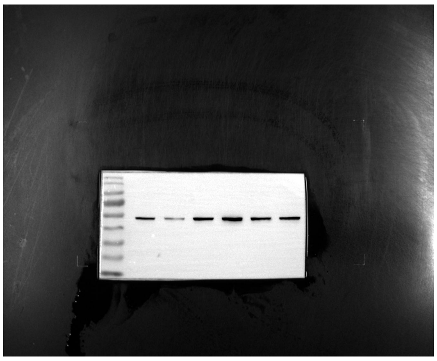

Beclin1

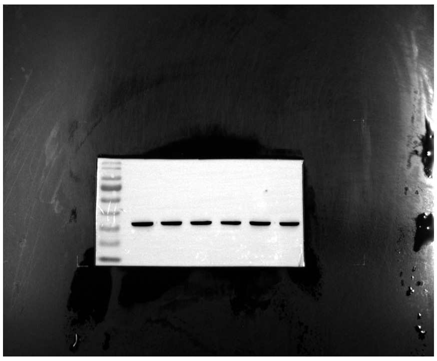

GAPDH

Figure 5B

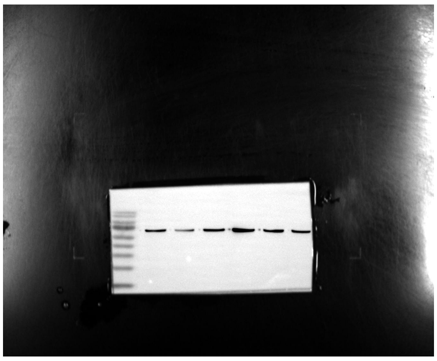

PINK1

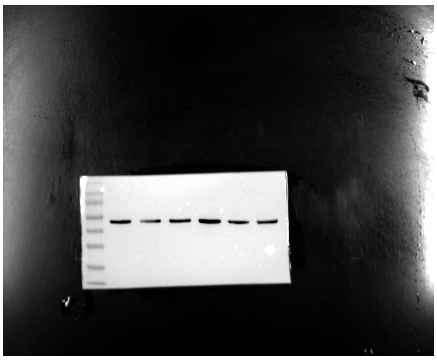

Parkin

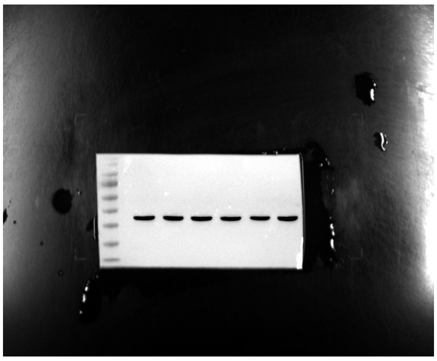

GAPDH

Figure 5C

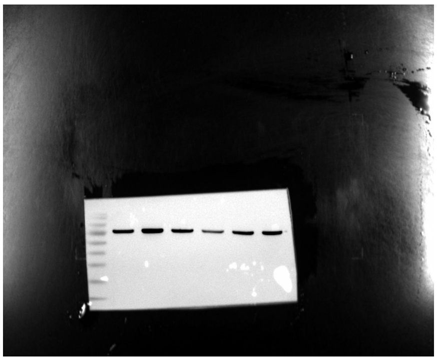

Mfn1

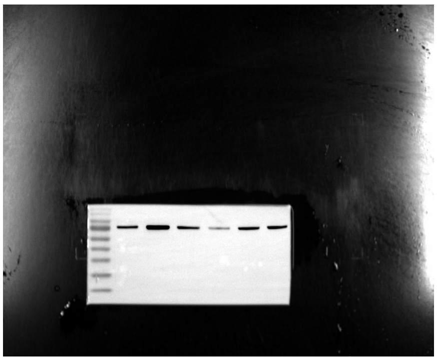

Mfn2

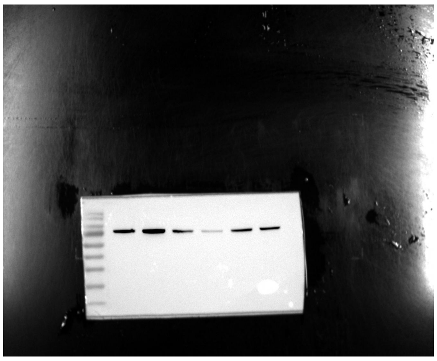

OPA1

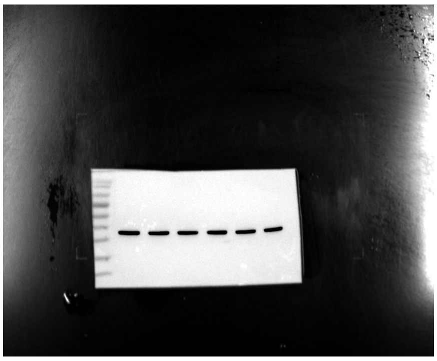

GAPDH

Figure 5H

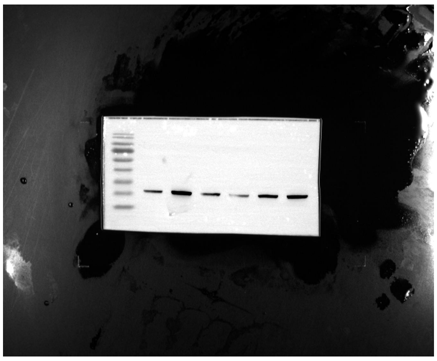

Bcl-2

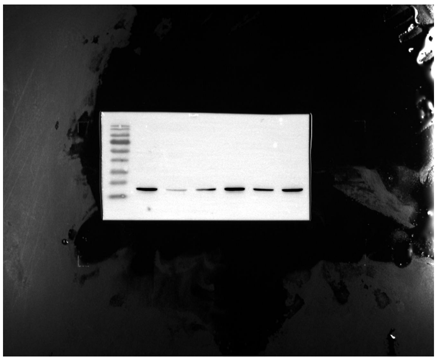

Bax

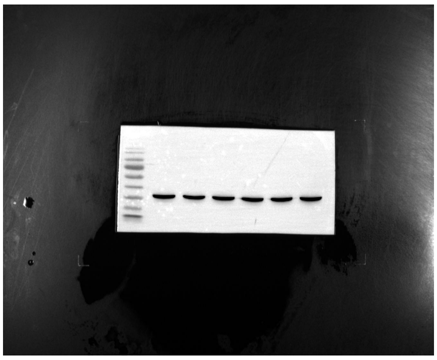

GAPDH
